# Influence of Nanobubbles on the Early Stages of Calcium Carbonate Formation

**DOI:** 10.1021/acs.cgd.5c00286

**Published:** 2025-09-03

**Authors:** Talie Zarei, Elmar C. Fuchs, Luewton L. F. Agostinho, Denis Gebauer, Jakob Woisetschläger, Herman L. Offerhaus

**Affiliations:** † 361182 Wetsus—Centre of Excellence for Sustainable Water Technology, Leeuwarden 8911 MA, The Netherlands; ‡ Optical Sciences Group, Faculty of Science and Technology (TNW), 207105 University of Twente, Enschede 7500 AE, The Netherlands; § Water Technology Research Group, 84808 NHL Stenden University of Applied Sciences, Leeuwarden 8911 MA, The Netherlands; ∥ Institute of Inorganic Chemistry, 26555 Leibniz University Hannover, Hannover 30167, Germany; ⊥ Working Group Laser Optical Metrology, Institute for Thermal Turbomachinery and Machine Dynamics, 3230 Graz University of Technology, Graz 8010, Austria

## Abstract

The mineralization process of calcium carbonate (CaCO_3_) is essential in both environmental and industrial applications, since it is a ubiquitous biomineral and a primary component of scale deposits in hard water systems. In this study, we examine the influence of charged nanobubbles, generated through vortexing ionic solutions (bi-/carbonate buffer at pH 10) with rotating nonmagnetic and magnetic impeller, on the formation of amorphous CaCO_3_ (ACC). Multi-angle light scattering and zeta potential nanoparticle tracking analysis show that alternating magnetic fields influence the size and charge of nanobubbles created by shear forces in vortexing. The altered nanobubble populations delay the formation of solid ACC by inhibiting the aggregation and coalescence of dense liquid calcium carbonate. We propose colloidal stabilization of the dense liquid intermediate by the nanobubbles along the pathway to solidification. Our findings suggest that magnetically manipulating nanobubble populations is a novel approach to controlling mineral scaling and designing advanced materials. This work highlights the potential role of nanobubbles in affecting mineral formation processes and provides new insights into mineralization control through physical treatment methods.

## Graphical abstract

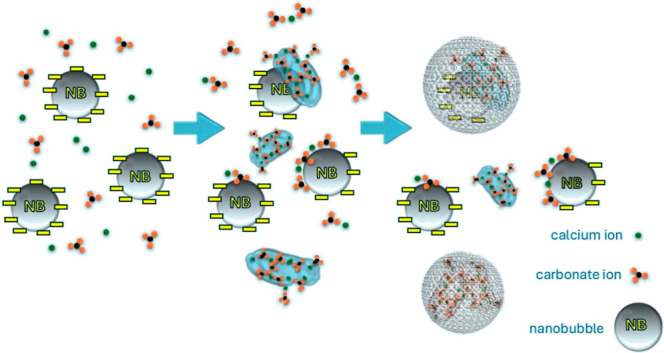

## Introduction

Calcium carbonate (CaCO_3_) nucleation and the study of nanobubbles represent two fascinating fields in materials science and physical chemistry that have recently begun to intersect, offering new insights into the crystallization processes, with potential applications in various industries. The formation mechanisms of calcium carbonate have long been a subject of intensive study due to its importance in biomineralization and industrial processes. Traditional views based on classical nucleation theory (CNT) propose a single-step process involving the direct formation of an amorphous or crystalline phase from a supersaturated solution.^1,2^ However, recent research has revealed a more complex, multistep process involving several intermediate stages^1,3–8^ including prenucleation clusters (PNCs), as small, thermodynamically stable clusters of calcium and carbonate ions that form even in undersaturated solutions;^9,10^ liquid-like precursor phases which represent a disordered, hydrated precursor phase that forms through liquid–liquid phase separation, including polymer-induced liquid precursors^11^ and dense liquid phases (DLPs);^12,13^ and amorphous calcium carbonate (ACC), a solid but disordered phase often preceding crystalline CaCO_3_ formation, with multiple types of ACC exhibiting different degrees of hydration^5,14^ as well as distinct short-range structures.^15^ Eventually, crystalline polymorphs as the final phases are formed, typically calcite, aragonite, or vaterite.^16^ Recent in situ observations using advanced microscopy techniques have provided direct evidence for nonclassical pathways: Cryo-TEM was used to visualize the early stages of calcium carbonate formation,^17^ while liquid-cell TEM has captured the aggregation and transformation of ACC precursors.^16,18^ These studies confirm the existence of multiple precursor species and pathways.

Nanobubbles are microscopic gaseous domains with diameters typically ranging from 50 to 200 nm.^19–27^ They have attracted significant attention due to their unique properties, including extraordinary stability,^20,28–30^ negative zeta potential,^20,31–38^ and high surface-to-volume ratio.^21–27^

The interaction between nanobubbles and calcium carbonate during nucleation and early growth stages is an emerging area of research. Several studies have begun to explore this intersection. Quach et al.^39^ investigated the effects of nanobubbles produced by an alternating magnetic field system on calcium carbonate solutions. Their nanoparticle tracking analysis (NTA) results suggested the formation of nanobubble–nanoparticle clusters, indicating a direct interaction between nanobubbles and calcium carbonate particles. Tagomori et al.^40^ studied the effects of air nanobubbles on calcite crystal growth. They observed that air nanobubbles could retard calcite crystal growth by up to 53% at 20 °C and 33% at 88 °C. A study by Fitzgerald et al.^41^ examined the interaction between nanobubbles and prenucleation calcium (-bi-) carbonate clusters during ultrasonic treatment of hard water. They found that PNCs could decorate nanobubbles in the early stages of sonication, suggesting a close interaction between these entities. Based on these studies, several hypotheses have emerged regarding how nanobubbles and calcium carbonate interact, including the following possibilities:

1. Nanobubble–nanoparticle clustering implying that nanobubbles may bind to calcium carbonate nanoparticles, nanodroplets, or PNCs, forming larger aggregates;^39,41^

2. Interfacial effects suggesting that the presence of nanobubbles may change the solid–liquid interfacial tension at crystal surfaces;^40^
3. An ion adsorption approach that indicates that the negatively charged surface of nanobubbles may adsorb calcium ions;^40^
4. Bubble mattress formation proposing that nanobubbles may form a “bubble mattress” on crystal surfaces;^40^
5. Local supersaturation effects pointing out that the high curvature of nanobubbles could create localized areas of high supersaturation near their surfaces^42^ and
6. Thermal effects highlight that nanobubbles may act as thermal buffers.^40^

While these studies have provided valuable insights, several key questions remain, such as

(a) The exact mechanism by which nanobubbles interact with PNCs, DLP, and ACC precursors during the early stages of nucleation,
(b) The influence of nanobubbles on the formation, stability, and transformation of liquid-like precursor phases,
(c) The role of nanobubbles in polymorph selection during CaCO_3_ crystallization,
(d) The effects of different nanobubble generation methods on CaCO_3_ nucleation and growth, and
(e) The potential for nanobubbles to control or modify CaCO_3_ crystallization pathways in biomimetic systems.

We consider both naturally occurring and experimentally enhanced presence of nanobubbles. A previous work has demonstrated that CO_2_ nanobubbles naturally exist in carbonate buffers at pH 10 ^42^ at low density.

We increased the nanobubbles’ number and charge density through nonmagnetic and magnetic vortexing cavitation. The comparison of the effects of native nanobubbles (as a reference) with those of enhanced populations and properties through vortexing treatments indicates that nanobubbles influence the early stages of ACC formation.

Static light scattering and zeta potential measurement were performed throughout the process to monitor the real-time size and charge evolution of the nanobubbles in the carbonate buffer and the prenucleation stages. Our findings indicate that these increased nanobubble populations delay the nucleation process by preventing dense liquid calcium carbonate aggregation and merging, ultimately slowing solid ACC formation. We suggest that this effect is due to colloidal stabilization of the dense liquid intermediate by the nanobubbles during the transition to solidification. The results suggest potential interactions between nanobubbles and mineralization intermediates, playing a role in influencing mineralization processes relevant to areas such as water hardness mitigation.

Despite recent advances, explicit experimental insights into how nanobubbles influence the early stage formation of amorphous calcium carbonate (ACC), particularly through nonclassical nucleation pathways, remain limited. Our work addresses this gap by systematically comparing naturally occurring nanobubbles with enhanced nanobubble populations generated through nonmagnetic and magnetic vortexing treatments. By employing detailed in situ analyses combining potentiometric titration, zeta potential nanoparticle tracking analysis (Z-NTA), and multiangle light scattering (MALS), we provide novel mechanistic insights into how magnetically manipulated nanobubbles kinetically stabilize dense liquid calcium carbonate intermediates, thereby delaying solid ACC nucleation. Furthermore, we elucidate the role of nanobubbles in modulating spinodal liquid–liquid phase separation processes, contributing deeper understanding of nonclassical nucleation mechanisms beyond CNT. These novel insights open new avenues for controlling mineral scaling and designing advanced materials through the physical manipulation of nanobubble populations.

## Materials and Methods

### Chemicals

Ultrapure water was used to prepare all solutions, including 50 mM CaCl_2_ (100.2%, VWR International, LLC.), 10 mM NaOH (diluted from the stock solution of 1 M, 99.9%, VWR International, LLC.), 10 mM NaHCO_3_ (99.7–100.3%, VWR International, LLC.), and 10 mM Na_2_CO_3_ (>99.5%, Sigma-Aldrich). The 10 mM carbonate buffer at pH 10 for each experiment was prepared freshly by adding the desired quantity of Na_2_CO_3_ to NaHCO_3_ while the pH was monitored online.

### Methods

#### Pretreatment of Carbonate Buffer

Two treated conditions were compared to a control, as given in Table 1, with at least five titrations per condition.

**Table 1:** Conditions of Control and Pre-treatment of Carbonate Buffer before the Titrations at pH 10

sample ID	condition	treatment
reference	control	
vortexed	treatment of carbonate buffer with a nonmagnetic impeller	five consecutive intermitted sessions of 50 s of nonmagnetic impeller vortexing at 600 rpm followed by a 10 s pause
magnetically vortexed	treatment of carbonate buffer with a magnetic impeller	five consecutive intermitted sessions of 50 s of alternating magnetic impeller vortexing at 600 rpm followed by a 10 s pause

For the control conditions, the carbonate buffer was prepared with fresh ultrapure water; no degassing procedure was applied to either the ultrapure water or the carbonate buffer. For the treatment, a polytetrafluoroethylene (PTFE) impeller was fabricated, as shown in Figure 1. Six holes at 60° intervals held either neodymium cylindrical disc magnets (NdFeB N45) or nonmagnetic stainless steel. The impeller was submerged 35 mm in water. The impeller was rotated at 600 rpm for 5 min during which hydrodynamic cavitation causes numerous microvortices.

**Figure 1:**
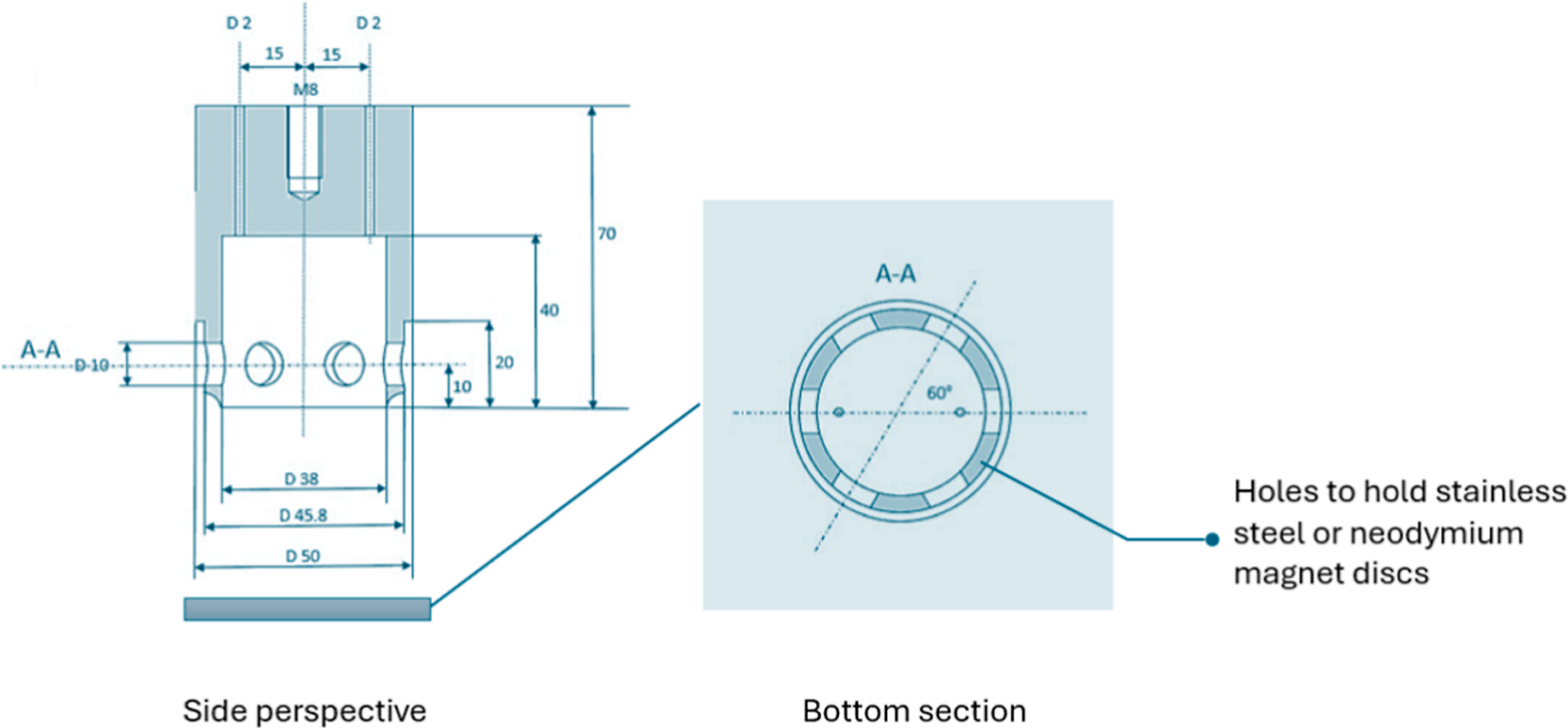
Schematic of the impeller used for pretreatment of carbonate buffer at pH 10 for both nonmagnetic and alternating magnetic conditions.

As summarized in Table 1, both nonmagnetic and magnetic vortexing treatments were performed under identical conditions, involving intermittent rotation cycles at 600 rpm for a total effective duration of 5 min. The impeller was used in a nonmagnetic and a magnetic configuration by embedding six stainless steel discs or six neodymium cylindrical disc magnets (NdFeB N45) at 60° angles to each other in the holes. In the magnetic configuration, the magnets were alternatingly oriented as S–N–S–N–S–N, creating six horizontal magnetic bottles around the impeller, as visualized in Figure 2.

**Figure 2:**
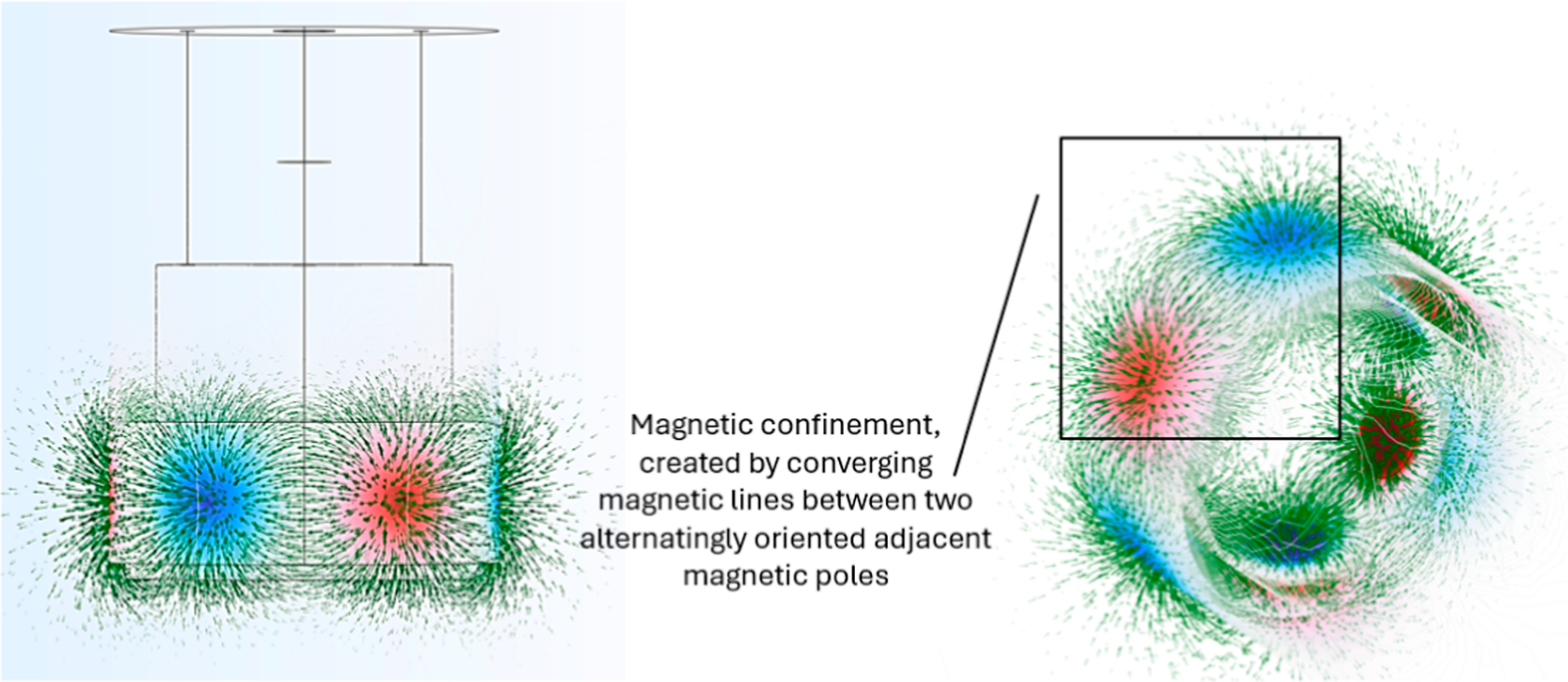
Magnetic flux at the horizontal plane of the impeller, visualized using COMSOL Multiphysics software.

After preparing the sodium bi-/carbonate buffer at pH 10, 80 mL was transferred to a lab beaker, and the impeller was placed inside of it so that the discs were submerged. After the treatment, the pH was monitored and adjusted at 10, if needed. Within less than a minute afterward, 50 mL was transferred to the vessel for the potentiometric titration. The temperature of the solutions was carefully monitored throughout all vortexing experiments and remained consistent at room temperature (22 ± 0.5 °C) for all treatments (native, vortex, and magnetically vortexed). Due to the moderate stirring speed (600 rpm), intermittent vortexing protocol (50 s rotation followed by 10 s pause), and relatively large solution volume (80 mL), no measurable temperature increases or significant differences between treatments were observed. Therefore, temperature effects were considered negligible and did not influence the observed differences in nucleation behavior.

#### Titration Procedure

Constant-pH titrations were automated using a method controlled by the software Tiamo 2.5. 50 mM CaCl_2_ was added at a rate of 0.05 mL min^–1^ (Metrohm, Titrando 888, exchange unit 806), and 10 mM NaOH (Metrohm, Titrando 905, dosing unit Dosino 800) was titrated automatically in order to maintain a constant pH of 10.0. Note that the addition rate in terms of mol/min was at least 25 times greater than in previous works using this methodology.^9^ We used a calcium-ion-selective electrode (Ca-ISE polymer membrane; Metrohm, No. 6.0508.110) and a pH electrode (Metrohm No. 6.0256.100), with the inner Ag/AgCl electrode of the pH electrode as the reference electrode. The pH electrode was calibrated weekly with standard buffers at pH values of 4.00, 7.00, and 9.00 (VWR International, LLC.). The Ca-ISE was calibrated daily by dosing 50 mM CaCl_2_ at a dosing rate of 0.05 mL min^–1^ to 50 mL ultrapure water at a preadjusted pH of 10 while keeping the pH constant by automatically adding 10 mM NaOH throughout the calibration up to the desired calcium potential for the experiments. During the titrations, the solution was evenly mixed with a poly­(tetrafluoroethylene) (PTFE) impeller at 270 rpm (R16, Ingenieurbüro CAT, M. Zipperer GmbH). Magnetic stirrers were avoided to exclude possible interferences with the experiment. The titrations were performed in a sealed quartz vessel. At the end of each titration, all surfaces in contact with the solutions, including the vessel, dosing tips, impeller, and electrodes, were washed with 10% CH_3_COOH or 3% HCl, rinsed adequately with ultrapure water, and dried using dust-free tissue paper.

#### Calculation of Free Calcium Ion Concentration

The calcium ion potential data *U*(Ca^2+^) was extracted from the ISE electrode measurement in order to calculate the molar concentration of free calcium ions, *c*(Ca^2+^). The concentration data was analytically calculated using the Nernst equation,^44^

1 $$U\left({Ca}^{2+}\right)=U_{0}+\frac{RT}{zF}\ln\left[\gamma_{app}\frac{c\left({Ca}^{2+}\right)}{c^{0}}\right]$$

where *U*_0_ is the electrode intercept. *R*, *T*, *z*, and *F* represent the gas constant, temperature, ion charge, and Faraday constant, respectively, γ_app_ is the apparent activity coefficient, and *c* ^0^ is the standard concentration (1 mol/L). Here, we assumed that γ_app_ = 1, that is, an ideal electrolyte, which was previously shown not to affect the evaluation significantly.^44^ The experimental electrode slope from calibration agreed with the theoretical value of (2.303 × *R* × *T*)/(2 × *F*), or 0.029587 V within experimental accuracy, but the calibrated electrode slope was used for data analyses regardless.

To determine the experimental ISE slope and intercept, the Ca^2+^ potential *U* gathered from the calibration was plotted as a function of log­[*c*(Ca^2+^)] and linearly fitted. The free calcium concentration *c*_free_ was then calculated as follows:

2 $$c_{free}\left({Ca}^{2+}\right)=\exp\left(\frac{U\left({Ca}^{2+}\right)-U_{0}}{a}\right)$$

where *a* is the experimental ISE slope.

#### Statistical Analysis of Potentiometric Titration Data

To minimize electromagnetic interference, the electrical cables of the Ca-ISE and pH electrodes were shielded with aluminum foil to protect the sensitive electrochemical measurements from external electromagnetic fields.

An overhead mechanical stirrer was used during the titration in order to avoid the influence of magnetic fields from the magnetic stirrers, especially in conjunction with vortexed and magnetically vortexed carbonate buffer.

The collected potentiometric titration data underwent thorough statistical analysis to ensure the reliability and validity of the results.

1. Identification of outliers: Outliers were identified based on deviations in the titration curves that significantly differed from the majority of the data. Criteria for outlier identification included the following:
  - Early or late nucleation: Curves showing nucleation occurring significantly earlier or later than the average, often in combination with other discrepancies in prenucleation slope or solubility threshold. There was approximately one such outlier in five repetitions, which is common for this type of experiments, due to, e.g., tiny impurities, etc.
  - Unusual fluctuations: Erratic “wiggles” or abrupt changes in potential not consistent with expected titration behavior indicting an instrumental issue.
  - For the calculation of solubility: Lack of plateau formation: Absence of a stable solubility plateau postnucleation.
2. Calculation of averages: After excluding outliers, averages were calculated as the following:
  - All titration curves were averaged to a common *x*-axis based on the cumulative amount of CaCl_2_ added to the maximum amount where all nonoutlier repetitions had data (i.e., up to 63 μmol of added Ca^2+^).
  - Determining solubility thresholds: The plateau regions following nucleation were identified for each replicate and gathered in a pool per experimental condition with a total of *N* > 100 data points, and the ion product (IP) values were then averaged across repetitions to obtain mean values for each experimental condition.

All raw data, including the excluded outliers, are provided in the Supporting Information, Section SI and Figures SI1, SI2.

#### Batch Multi-angle Static Light Scattering Analysis and Data Evaluation

In batch mode, multi-angle light scattering (MALS), DWAN 8 (Wyatt Technology Europe, GmbH), was used to investigate the size distribution of species occurring in the titration experiment. The samples were injected into the flow cell inside the MALS module at a flow rate of 0.5 mL min^–1^, where the liquid is exposed to the incident emission at 660 nm, θ = 0°, and the scattered light is collected by eight angularly positioned detectors from 32° to 141°. The scattering light is converted to voltage, proportional to the detectors’ normalization coefficients, and the root mean square (RMS) size is calculated according to eq 3,^45^

3 $$R=\sqrt{\frac{\sum m_{i}r_{i}^{2}}{\sum m_{i}}}$$

with *R*, *m*_*i*_, and *r*_*i*_ representing the RMS radius of the *i* ^th^ mass element from the gravity center and *i* ^th^ distance from it, and the number density of the nanoentities in the aqueous states prior to the sample analysis, ultrapure water was recorded as a baseline. After that, an amount of sample was injected sufficient to condition the flow cell and stabilize the signal before recording the light scattering for 5 min, followed by a cleaning sequence consisting of ultrapure water (5 min at 1 mL min^–1^), 0.1% formic acid (1 min at 1 mL min^–1^), and ultrapure water (20 min at 1 mL min^–1^), followed by ultrasonic cleaning for the flow cell (10 min) when necessary depending on the signal level, noise, and wander characteristics of ultrapure water. Each sample was prepared and analyzed three times. After the analysis, the light-scattering data was processed using ASTRA 8.0.0.19 software (Wyatt Technology, Europe). Different form factors were applied to the light scattering signals for the size evaluation, enabling specific detector signals to compare the possible fittings. The shape-derivative formalism Sphere (eq 4)^45,46^ was found to be the best fit from which the average RMS radius and number density were extracted,

4 $$P\left(\theta \right)={\left[\left(\frac{3}{x^{3}}\right)\left(\sin x-x\cos x\right)\right]}^{2},,x=\frac{2\pi D}{\lambda }\sin\left(\frac{\theta }{2}\right)$$

with *P* signifying the light scattering intensity dependence on the angle θ and *D* and λ represent the sphere’s diameter and the incident light’s wavelength, respectively. In this formalism, the nanoentities are assumed to be spherical. The data extracted for each sample was aggregated for subsequent statistical analysis.

An overview of the different samples analyzed with batch MALS is given in Table 2.

**Table 2:** Carbonate Buffer and the Pre-nucleation Samples during Titrations for All Three Conditions, Including Control, Non-magnetic, and Magnetic Impeller Treated, Analyzed Immediately after Their Preparation with MALS in Batch Mode, with Three Repetitions for Each Sample

sample	conditions	concentrations
carbonate buffer at pH 10	reference, vortexed, and magnetically vortexed	10 mM
prenucleation solutions during the titration	reference, vortexed, and magnetically vortexed	point 1: 0.15 mL of CaCl_2_ addition
		point 2: 0.45 mL of CaCl_2_ addition
		point 3: 0.65 mL of CaCl_2_ addition

#### Z-NTA

To determine the zeta potential and hydrodynamic diameter, the same samples listed in Table 2, except for point 2, were also injected into Z-NTA, in parallel to the MALS. Similarly, three repetitions were analyzed to ensure data reproducibility. Measurements were conducted using the NanoSight NS500 instrument (Malvern Panalytical, UK) that uses NTA, an image-processing technique based on the real-time microscopic visualization of individual particles undergoing Brownian motion. Typically, particle movements are recorded over periods of about 90 s. Their translational diffusion coefficients are calculated by tracking each particle’s movement. The hydrodynamic diameter *d* of each particle is then determined using the Stokes–Einstein equation:

5 $$D_{t}=\frac{TK_{B}}{3\pi \eta d}$$

where *D*_t_ is the translational diffusion coefficient, *T* is the absolute temperature of the sample, *K*_B_ is the Boltzmann constant, and η is the viscosity of the solvent.

The data collected from each sample’s three repetitions were gathered in one data pool and presented as the median and interquartile range. Additionally, the hydrodynamic diameter resulting from the NTA is provided in the Supporting Information file, Section SV, Figure SI5.

## Results and Discussion

Figure 3a shows the evolution of the free calcium ion concentration as a function of the amount of CaCl_2_ added to the carbonate buffer at pH 10. Figure 3b compares the ion products of free calcium and carbonate concentrations per conditions over the cumulative amount of added CaCl_2_. The averaged solubility thresholds calculated, as explained earlier in the section on Methods, are added to the graph as an inset.

**Figure 3:**
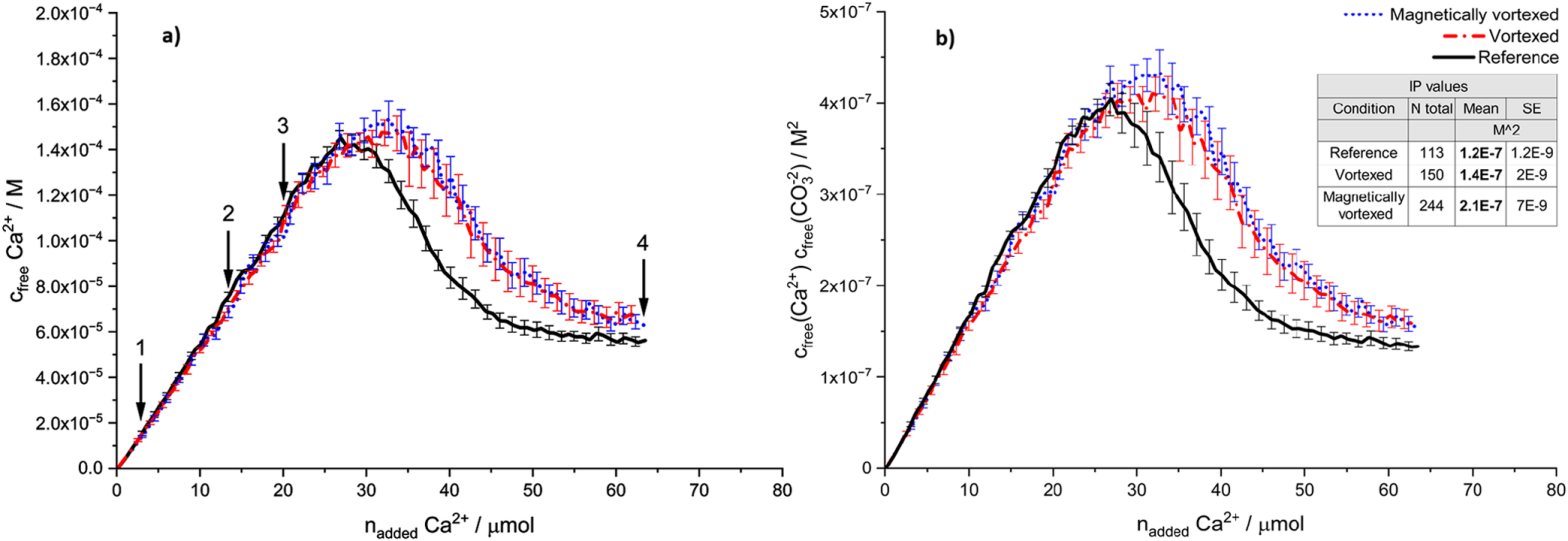
Results from potentiometric titrations at pH 10 for three conditions of reference (*N* = 5), vortexed (*N* = 7), and magnetically vortexed (*N* = 11): (a) Free calcium ion concentration over the amount of added CaCl_2_. Higher free calcium ions are needed to reach the nucleation, and delayed nucleation after treatments is evident. Within experimental certainty, the prenucleation slopes are not affected by the different conditions. The curves represent the mean ± standard error of mean to compare the means of the three conditions. The error bars are reduced to a portion of the total data points for better readability. Arrows relate to the sample collection points for MALS (1,2,3), Z-NTA (1,3), scanning electron microscopy (SEM) (4), and FTIR (4) analyses throughout the titrations. SEM and FTIR results are presented in the Supporting Information file, Sections SII–SIV; (b) free IP averaged per condition over the added CaCl_2_. Initial ACC formation shows an elevated state after the treatments, as reflected in slightly higher solubility thresholds provided in the inset. These thresholds were gathered and averaged from the plateau regions of each individual replicate per condition. The data represent the mean ± standard error of the mean.

In Figure 3, the potentiometric titrations show that the free calcium ion concentration at the nucleation point is slightly higher in the treated samples (vortexed and magnetically vortexed) than in the reference. This shows that more calcium ions were added to the solution before nucleation occurred, implicating delayed nucleation and a higher level of supersaturation before nucleation. While Figure 3a illustrates the kinetic influence of nanobubbles by showing delayed nucleation (higher free calcium concentration at nucleation), Figure 3b complements this by emphasizing the thermodynamic aspects through the IP of calcium and carbonate ions. Specifically, Figure 3b quantitatively demonstrates how enhanced nanobubble populations allow the system to reach higher supersaturation levels prior to nucleation, reflecting the thermodynamic stabilization of dense liquid calcium carbonate intermediates. Thus, the kinetic delay in nucleation (Figure 3a) is accompanied by a measurable increase in supersaturation (Figure 3b), jointly confirming the significant influence of nanobubbles on both the kinetic and thermodynamic aspects of ACC nucleation. It has been suggested that nanobubbles delay nucleation through several potential mechanisms.^39–42^ Our findings suggest that they may prevent ion collisions or cluster interactions by acting as barriers, delaying the aggregation of the DLP necessary for nucleation to proceed. Although these differences are modest and should be interpreted cautiously, they suggest that nanobubbles might kinetically stabilize the dense liquid calcium carbonate phase, delaying its transformation into solid amorphous calcium carbonate.^47^ From the viewpoint of CNT, on the other hand, it is hard to rationalize an inhibition of nucleation in the presence of charged solid/liquid interfaces introduced by the nanobubbles, as such effects commonly lead to increased nucleation rates in heterogeneous nucleation scenarios.

The solubility thresholds calculated from the plateau regions of the titration curves provide further insight into these observations. The average solubility product (*K*_sp_) values for the different conditions are found to be as follows:

- Reference: *K*_sp_ = 1.2 × 10^–7^ ± 1.2 × 10^–9^
- Vortexed: *K*_sp_ = 1.4 × 10^–7^ ± 2 × 10^–9^
- Magnetically vortexed: *K*_sp_ = 2.1 × 10^–7^ ± 7 × 10^–9^

These elevated solubility thresholds in the treated samples, especially after the magnetically vortexed treatment (see Figure 3b inset), support the notion that nanobubbles influence the nucleation process beyond the kinetic stabilization of dense liquid intermediates. In fact, the ACC formed exhibits a somewhat higher solubility corresponding to a thermodynamic destabilization, which might be due to the incorporation of nanobubbles, higher water contents, and/or a higher degree of structural disorder within the solid ACC. However, given the experimental variability, these findings should be considered indicative, and further results from MALS and Zeta potential measurements are warranted to confirm the nanobubbles’ influence.

A previous work^48^ showed that the spinodal regime for liquid–liquid demixing toward dense liquid calcium carbonate formation can be reached upon increasing the mixing rate of calcium and carbonate precursor solutions. Our data, in fact, indicate that the ACC obtained here corresponds to the theoretical solubility threshold predicted for that process, within experimental accuracy. Additionally, the nanobubble populations can partially inhibit the processes from spinodal dense liquid (SDLP) calcium carbonate to solid ACC, i.e., aggregation and coalescence of the SDLP, and its dehydration/solidification. This effect is reflected in the delayed onset of nucleation of ACC as discussed above, while the elevated solubility thresholds, as mentioned above in the inset of Figure 3b, indicate a thermodynamic destabilization of the as-formed ACC by nanobubbles. In order to obtain further insight into the proposed mechanism, MALS analyses were performed.

Figure 4a–d shows the light scattering signal collected from the batch MALS analysis of the reference, vortexed, and magnetically vortexed conditions from the carbonate buffer (Figure 4a) and sample collection points 1 (after 0.15 mL of added CaCl_2_, in the undersaturated state) (Figure 4b), 2 (after 0.45 mL of added CaCl_2_, after the solubility level) (Figure 4c), and 3 (after 0.65 mL of added CaCl_2_, in the supersaturated state, before the nucleation of solid ACC) (Figure 4d). Three repetitions are presented as three sets in a continuous recording signal with the ultrapure water (solvent) as the baseline. Figure 4e presents the nanobubbles’ average RMS size (nm) associated with the MALS signals for all samples, while Figure 4f excludes the results from the sample collected above the solubility level, showing visible trends in the size evolution of nanobubbles from the initial state in the buffer to the titrations at undersaturated and supersaturated (before the nucleation) states. Note that point 2 (at 0.45 mL of CaCl_2_ addition, slightly above the solubility threshold) represents a transient state characterized by pronounced heterogeneity and polydispersity due to the onset of liquid–liquid phase separation. At this transitional stage, significant fluctuations obscure clear interpretation. Therefore, to better illustrate clearer and more stable trends in nanobubble size evolution, we excluded point 2 from Figure 4f and focused instead on the stable states provided by the carbonate buffer, undersaturation (point 1), and supersaturation immediately prior to nucleation (point 3).

**Figure 4:**
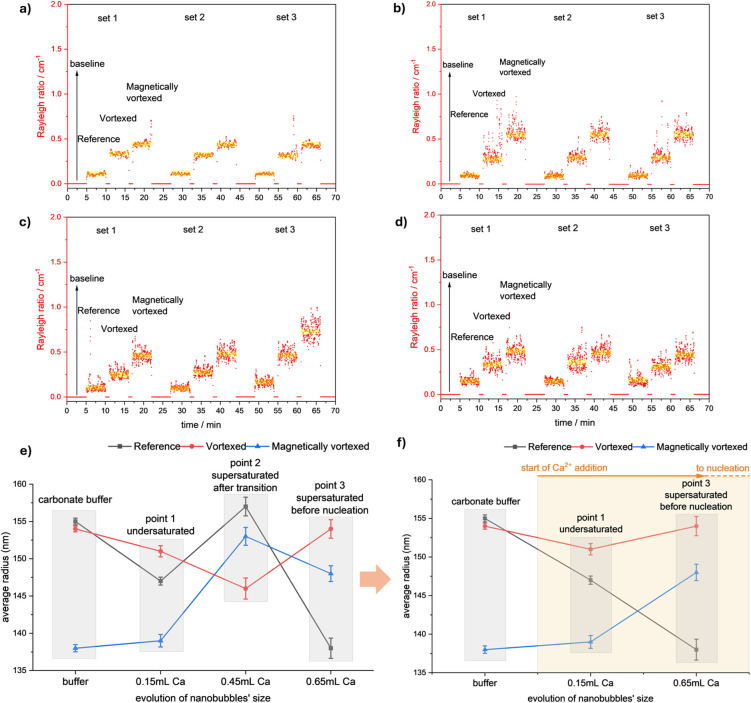
Light scattering of the reference, vortexed, and magnetically vortexed conditions in (a) carbonate buffer, (b) point 1 of undersaturation, (c) point 2 of supersaturation above the solubility level, and (d) point 3 of supersaturation before the nucleation; all in three repetitions with the solvent (ultrapure water) as the baseline. The nanobubbles’ average RMS size (nm) extracted from the MALS signals (highlighted in yellow) for (e) all the samples and (f) excluding point 2 of supersaturation above the transition, a trend of decrease, monotonous, and increase in nanobubbles’ size change can be followed for each experimental condition of reference, vortexed, and magnetically vortexed treatments, respectively. Error bars represent ± 1-σ-standard of error of the mean.

The Rayleigh ratio intensity on the *y*-axis of Figure 4a–d, proportional to the size distribution and concentration of scatterers in the solution, highlights three important points: first, the number of native (in reference condition) and induced (in vortexed and magnetically vortexed conditions) nanobubbles remains relatively intact among the mother analyte (carbonate buffer) and the undersaturated and supersaturated states, and there is no measurable number density change like coalescence in the nanobubbles’ population during the potentiometric titration involving stirring at 270 rpm. Although direct experimental quantification of nanobubble number densities was not performed in our study, recent literature provides clear reference values. Zarei et al.^43^ explicitly quantified intrinsic nanobubbles in alkaline solutions at similar pH (∼10) using field-flow fractionation multi-angle light scattering and NTA. They reported number densities of approximately 10^4^ to 10^6^ nanobubbles per milliliter in carbonate buffer solutions under comparable conditions. Therefore, the observed Rayleigh intensity variations in our current study (Figure 4a–d) are consistent and can be reasonably attributed to differences in nanobubble number density, in agreement with these literature values. Second, the Rayleigh ratio intensity shows increasing signal fluctuations from carbonate buffer (Figure 4a) through undersaturation (Figure 4b) to supersaturation levels (Figure 4c,d) approaching nucleation. This increase in signal noise can be explained as follows: it has been shown that^47–49^ the aqueous calcium carbonate solution undergoes liquid–liquid phase separation as the solubility threshold (also corresponding to the liquid–liquid binodal limit) of proto-structured ACC is exceeded (ca. 4 × 10^–8^ to 5 × 10^–8^ M^2^, depending on the specific pH). Here, owing to the high addition rate and large concentration gradients, even the spinodal limit (∼10^–7^ M^2^) can be readily exceeded, yielding ACC with significantly increased solubility when compared to proto-structured, “binodal”, ACC. In any case, upon liquid–liquid separation, the environment becomes chemically and physically more heterogeneous as supersaturation increases due to increased concentrations of PNCs and dense liquid nanodroplets, alongside a mixture of nanobubbles. These conditions can lead to localized changes in refractive index or physical properties like viscosity. In addition, this increase in the noise could correspond to the evolution from a monodisperse population of nanobubbles to a polydisperse mixture of varying sizes. Furthermore, although PNCs and initially formed dense liquid nanodroplets are below the direct size detection limit of MALS due to their small sizes (1–3 nm), their dynamic behavior significantly impacts the scattering signal. As the dense liquid nanodroplets aggregate, coalesce, and/or undergo dehydration/solidification toward amorphous calcium carbonate (ACC), they cause fluctuations in the solution’s refractive index. In a monodisperse system, particles of similar size scatter light uniformly, resulting in a smoother and high-resolution signal. Conversely, in a polydisperse system, the presence of particles of different sizes leads to varying scattering contributions at each angle, causing fluctuations in the measured intensity over time. The observed increase in noise is also reflected in the higher uncertainty of the average size, leading to the increasing trend in the standard error presented in Figure 4e,f. By considering the contributions of liquid–liquid phase separation in the aqueous calcium carbonate system, one can obtain a more comprehensive understanding of the factors influencing the scattering behavior and noise in the measurements.

In Figure 4e, nanobubbles’ radii from point 2 appear to deviate from the other conditions. The deviation suggests that a distinct process might occur at this threshold. These processes could increase polydispersity and heterogeneity at this stage in the average particle size detected by MALS.

In Figure 4f, by excluding the data from point 2, we can envision a trend in the nanobubbles’ average size changes as decreasing for the reference and increasing for the magnetically vortexed sample. Magnetic vortexing initially produces the smallest nanobubbles, and these nanobubbles exhibit a more notable increase in size during supersaturation. This higher growth rate could be attributed to their larger surface-area-to-volume ratio, which may enhance interactions with ions, clusters, dense liquid calcium carbonate nanodroplets, or other species in the solution.

Figure 5 shows the overall measurements of the zeta potential for the reference, vortexed, and magnetically vortexed conditions from the carbonate buffer, point 1, and point 3.

**Figure 5:**
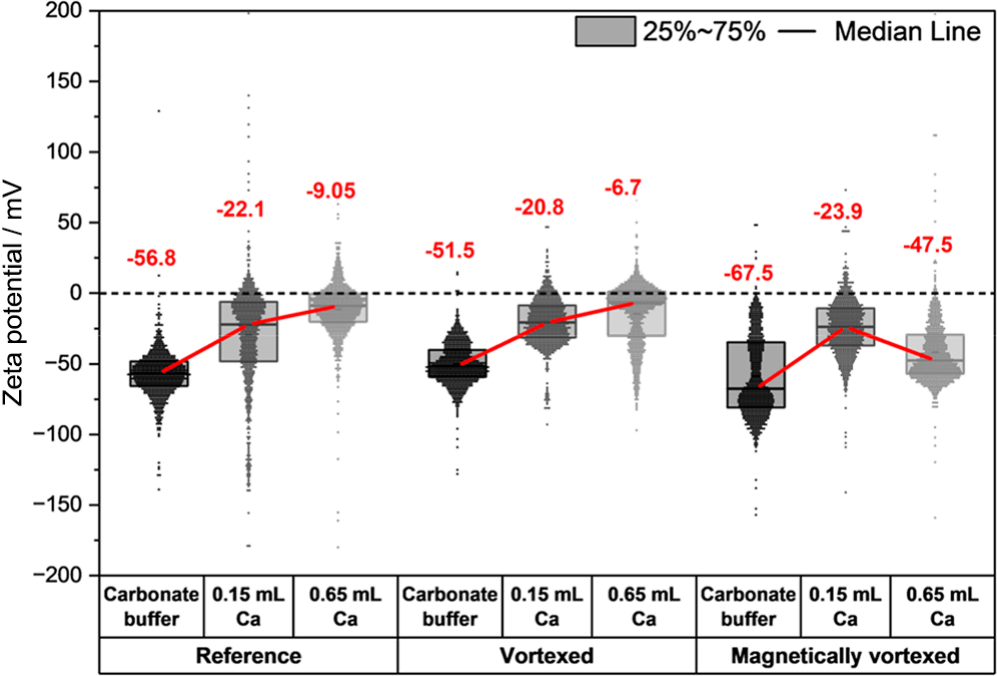
Zeta potential distributions (mV) for three experimental groups of reference, vortexed and magnetically vortexed from the carbonate buffer, point 1 as the undersaturated sample after the addition of 0.15 mL of Ca addition, and point 3 as the supersaturated sample with 0.65 mL of Ca added to the carbonate buffer. The red solid line connects the medians to show the trend in the nanobubbles’ zeta potential evolution from the carbonate buffer toward the nucleation.

Figure 5 shows that initially, all native and induced nanobubbles exhibited negative zeta potentials, specifically −57 mV for the reference, −52 mV for the vortexed, and −68 mV for the magnetically vortexed samples, indicating stable, negatively charged nanobubbles due to the preferential adsorption of hydroxide ions at their surfaces, in agreement with what was previously reported.^20^

The change of nanobubbles’ zeta potential from carbonate buffer toward the nucleation of solid ACC demonstrates that upon the addition of calcium ions (Ca^2+^), the bubbles in all samples became less negative, converging to approximately −22 mV, regardless of the initial condition. The added ions create an electric layer of cations underneath the negative bubble surface underneath. This change suggests that Ca^2+^ ions adsorbed onto the negatively charged nanobubble surfaces, partially neutralizing their charge. However, no significant additional binding of calcium ions becomes obvious in the titrations and potentiometric measurements (Figure 3a). Note that the measured free Ca^2+^ concentration (Figure 3a) is consistent with literature data on PNC formation; i.e., a significant fraction of calcium and carbonate ions is bound in these ion associates. Since no additional binding is observed in the presence of bubbles, interactions of calcium ions with nanobubble are, if any, very weak. While reversible ion adsorption–desorption equilibria are common in colloidal and interfacial systems,^4,9,32,34^ the measurements would reveal the ergodic average of that process even if it was highly dynamic.

Different behaviors can be detected when progressing to the supersaturation stage just before the formation of solid ACC. The zeta potential continuously decreases for the reference and vortexed nanobubbles. However, as mentioned above, since our data does not show significant additional Ca^2+^ adsorption at this stage (Figure 3a), this change might not be due to continued Ca^2+^ adsorption. Still, it could be attributed to the binding of nanobubbles to larger species such as CaCO_3_ clusters and droplets. This interaction may preferentially remove the more negatively charged nanobubbles from the population, resulting in a less negative average zeta potential for the remaining nanobubbles. This trend can destabilize the nanobubbles, possibly favoring nucleation as the electrostatic repulsion diminishes. Figure 6 illustrates the proposed mechanism of the nanobubbles’ effect on calcium carbonate nucleation.

**Figure 6:**
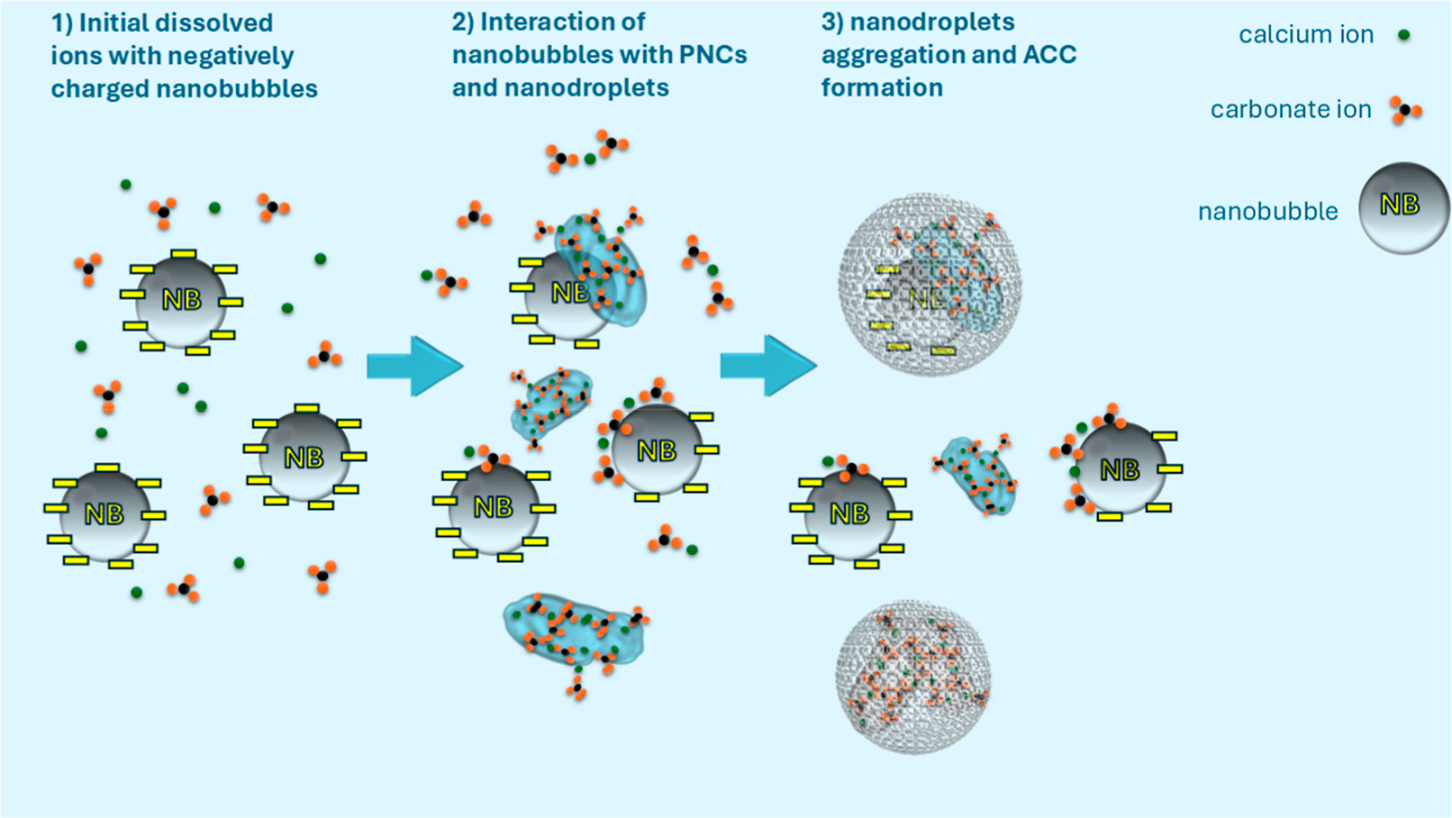
Schematic representation of the influence of nanobubbles on the early stages of calcium carbonate formation. (1) In the carbonate buffer at pH 10, negatively charged nanobubbles coexist with free calcium and carbonate ions. (2) PNCs and nanodroplets form near the nanobubbles; they might adsorb onto the nanobubble surfaces. Nanobubbles stabilize dense liquid calcium carbonate nanodroplets, preventing their aggregation. (3) As a result, the formation of solid amorphous calcium carbonate (ACC) is delayed. The presence of nanobubbles leads to a kinetic stabilization of the dense liquid state, and thermodynamic destabilization of ACC, likely due to the incorporation of nanobubbles and a higher structural disorder of the resulting solid.

The significant decrease in zeta potential that we observe for the magnetically vortexed solutions suggests that the induced nanobubbles, which are smaller, higher in number, and stronger negatively charged, may preferentially adsorb more anions like carbonate or bicarbonate ions in a second layer with positive charge in between, enhancing their negative surface charge. Additionally, this reversal may be explained by the formation and adsorption of dense liquid CaCO_3_ nanodroplets or prenucleation species.^4,44^ At this stage, the nanobubble surfaces may become decorated with these species, restoring a more negative zeta potential. This scenario aligns well with the principles of nonclassical nucleation theory, indicating the presence and adsorption of negatively charged CaCO_3_ nanodroplets onto nanobubble surfaces at higher supersaturation levels.

This effect could increase electrostatic repulsion, stabilize dense liquid CaCO_3_ nanoentities, and prevent coalescence. Consequently, by colloidally stabilizing the dense liquid CaCO_3_ phase, the nanobubbles delay its transformation into solid ACC. This is also in line with the higher solubility of the formed ACC, indicating a higher thermodynamic metastability; due to the inclusion of nanodroplets, the ACC could be more disordered and retain more water and other defects, rendering it somewhat more soluble than without the colloidal stabilization of the dense liquid precursor. Similar observations^50^ were made in the presence of certain polymers capable of stabilizing dense liquid calcium carbonate.

Analysis of the precipitated solids by attenuated total reflection Fourier-transform infrared (ATR-FTIR) spectroscopy (Figure SI3) and SEM imaging (Figure SI4) confirms that the primary crystalline polymorph formed under all experimental conditions is calcite, accompanied by a minor presence of vaterite, indicated by a characteristic FTIR band at 744 cm^–1^. SEM images consistently reveal dominant rhombohedral calcite morphologies, alongside occasional spherical morphologies potentially indicative of vaterite particles. These results indicate that while vortexing and magnetically vortexing treatments significantly influence early stage nucleation processes by stabilizing dense liquid calcium carbonate intermediates, the final crystalline polymorph distribution remains essentially unaffected. Such polymorph mixtures (calcite and minor vaterite) are commonly observed under similar experimental conditions.^4,5^ Future investigations employing cryogenic transmission electron microscopy could directly visualize the hypothesized dense-liquid calcium carbonate intermediates and possible hollow ACC structures formed around nanodroplets, further confirming the proposed stabilization mechanism suggested by our current findings.

## Conclusions

Our study investigated the impact of nanobubbles on the early stages of calcium carbonate (CaCO_3_) formation in carbonate buffer solutions at pH 10, considering naturally occurring nanobubbles and those enhanced through vortexing and magnetic vortexing treatments. By employing potentiometric titrations, zeta potential measurements, and MALS, we explored how changes in nanobubble populations and properties influence the nucleation process of the initially formed solid ACC.

Considering the conditions of our experiments and recent findings in the literature, it appears that we have induced the formation of amorphous calcium carbonate (ACC) via spinodal liquid–liquid demixing and subsequent dehydration/solidification rather than classical nucleation pathways. In particular, the study by Avaro et al.^48^ demonstrated that stable prenucleation calcium carbonate clusters define liquid–liquid phase separation, leading to ACC formation through spinodal decomposition when high mixing rates of the precursor solutions allow reaching IP values on the order of ∼10^–7^ M^2^. In our experiments, the significantly increased addition rate of calcium ions—using 50 mM CaCl_2_ at 0.05 mL min^–1^, representing a 25-fold increase in molar addition rate compared to the standard 10 mM CaCl_2_ at 0.01 mL min^–1^—resulted in rapidly elevated supersaturation levels. This rapid increase drives the system into the spinodal regime, where phase separation occurs spontaneously without the need to overcome the energy barrier associated with nucleation.

The influence of nanobubbles on this process is particularly noteworthy. The nanobubbles generated, especially through magnetic vortexing, are smaller and more numerous and exhibit a higher negative surface charge. These characteristics enhance their ability to interact with calcium carbonate species in solution. The nanobubbles may stabilize the dense liquid calcium carbonate phase by adsorbing on their interfaces and preventing their coalescence into larger aggregates occurring on the pathway to solid ACC. This colloidal stabilization effect delays the transformation of the DLP into solid ACC subsequent to spinodal decomposition. Induced nanobubbles, particularly the magnetically vortexed ones, delay calcium carbonate formation by stabilizing an intermediate dense liquid calcium carbonate phase and delaying its transformation into solid ACC. This inhibition is evidenced by the higher free calcium ion concentrations and IP values required for nucleation in the treated samples. We propose that negative charges lead to interactions with the dense liquid calcium carbonate nanodroplets, colloidally stabilizing the nanobubbles and preventing aggregation necessary for the formation of solid ACC. Nanobubbles may attract and bind CaCO_3_ clusters and droplets. Our findings from potentiometric titrations, zeta potential measurements, and MALS support the conclusion that nanobubbles influence the dynamics of the process from liquid–liquid separation toward solid ACC. The increased signal fluctuations and polydispersity observed in MALS near ACC formation further emphasize the role of nanobubbles in altering solution dynamics and influencing liquid–liquid separation in the aqueous calcium carbonate system. However, nanobubbles not only influence the kinetics of nucleation and growth processes but also appear capable of altering ACC’s thermodynamic solubility. Specifically, the presence of nanobubbles seems to increase the solubility of ACC, as suggested by the solubility products. This enhancement in solubility suggests that nanobubbles may destabilize ACC. Thus, the presence of nanobubbles impacts both the thermodynamics and the kinetics of the system. However, nanobubbles do not seem to alter the calcium carbonate crystalline phase, as confirmed by the results extracted from SEM and ATR-FTIR (presented in the Supporting Information, Sections SII and SIII, respectively), showing that the final crystalline products remained consistent across treatments despite differences in the initial nanobubble conditions (native or induced), corroborating the hypothesis that the treatment is merely an amplification of the natural conditions—nanobubbles are present in all cases; the physical treatment only changes their charge and number density.

Understanding how nanobubbles inhibit solid formation enables new strategies to prevent industrial-scale formation. Manipulating nanobubble populations and properties offers the potential for controlling crystallization processes in materials science. The study provides valuable insights into natural biomineralization processes, where ACC is a transient precursor. The effects of magnetic vortexing on the nanobubble behavior highlight the potential impact of magnetic fields in water treatment technologies aimed at mitigating water hardness.

By integration of findings from multiple analytical approaches, our study underscores the potential influence of nanobubbles on calcium carbonate nucleation. The induced nanobubbles, especially magnetically manipulated, alter the nucleation landscape by inhibiting the growth and ripening of the initially dense liquid calcium carbonate toward solid amorphous particles. These insights open avenues for controlling mineral formation processes across various scientific and engineering disciplines, contributing to advancements in scale prevention, material design, and understanding of biomineralization.

## Supplementary Material



## Data Availability

The data supporting the plots in this paper and other findings of this study are available from the corresponding authors upon request.

## Notes

Detailed data on the potentiometric titrations, SEM and ATR-FTIR investigations, analyses of ATR-FTIR spectra and SEM images, and discussion of the hydrodynamic diameter extracted from Z-NTA measurements (PDF)
