# Supplementary material for: Influence of Nanobubbles on the Early Stages of Calcium Carbonate Formation
Source: Cryst Growth Des. 2025 Sep 3;25(18):7417–27. doi: 10.1021/acs.cgd.5c00286 (PMC12447490; doi:10.1021/acs.cgd.5c00286)
Supplement: Supplementary file 1 [file cg5c00286_si_001.pdf]

Influence of Nanobubbles on the Early Stages of Calcium Carbonate Formation

Talie Zarei[1,2], Elmar C. Fuchs[1,2], Luewton L.F. Agostinho[1,3], Denis Gebauer[4], Jakob Woisetschläger[5] and Herman L. Offerhaus[2]

[1] Wetsus - Centre of Excellence for Sustainable Water Technology, Leeuwarden, the Netherlands

[2] Optical Sciences Group, Faculty of Science and Technology (TNW), University of Twente, Enschede, the Netherlands
[3] Water Technology Research Group, NHL Stenden University of Applied Sciences, Leeuwarden, the Netherlands
[4] Leibniz University Hannover, Institute of Inorganic Chemistry, Hannover, Germany
[5] Working Group Laser Optical Metrology, Institute for Thermal Turbomachinery and Machine Dynamics, Graz University of Technology, Graz, Austria

Supplementary information

I) Potentiometric titrations

In our potentiometric titration experiments, several data traces exhibited anomalies such as early or delayed nucleation and erratic fluctuations in potential. Given that these factors can introduce significant errors not representative of the system's true behavior, the affected data points were excluded from the final analysis in the main results. In the following, the concentration of free calcium ions as a function of the total amount of calcium is shown with detailed replicates excluding (on the left) and including (on the right) the outliers. The outliers are labeled on the graphs. The average curves (solid red line) are calculated on the common X-axis.

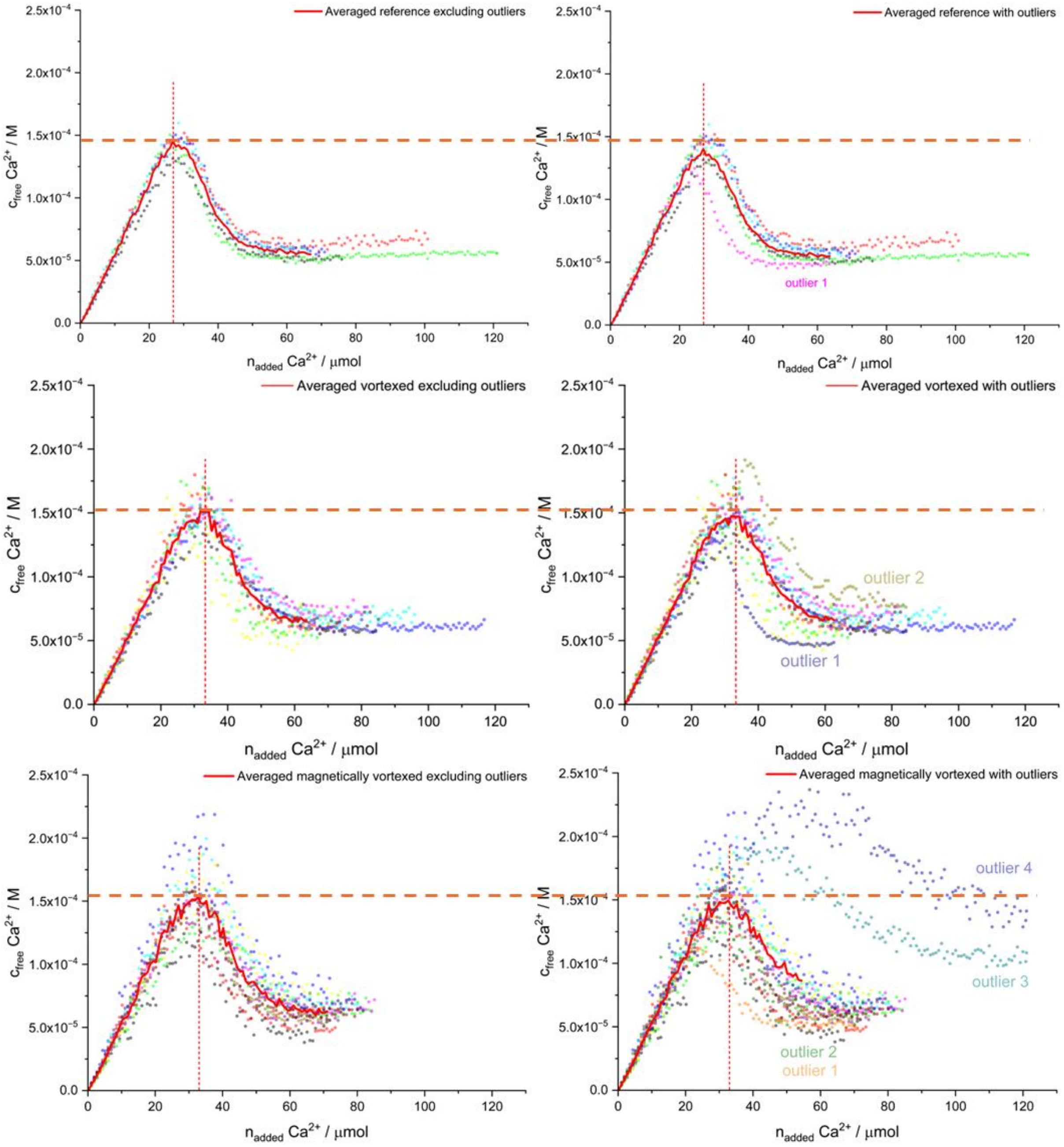

Figure SI 1. Free calcium ion concentration against the amount of added $CaCl_2$ at pH 10 was extracted from the potentiometric titration curve recorded by Ca-ISE for the three conditions of reference, vortexed, and magnetically vortexed. The curves represented with dot symbols are the replicates and the solid red line, the average of the three conditions, considering the outliers affecting the average curve. The outliers on the right-handed graphs are labeled within each condition.

Figure SI2 shows the replicates of the ion products of the replicates under the three conditions of reference, vortexed, and magnetically vortexed over the added amount of calcium, considering the outliers. Here, similar to Figure SI1, a decrease in the maximum of the average curves calculated, including the outliers, can be seen.

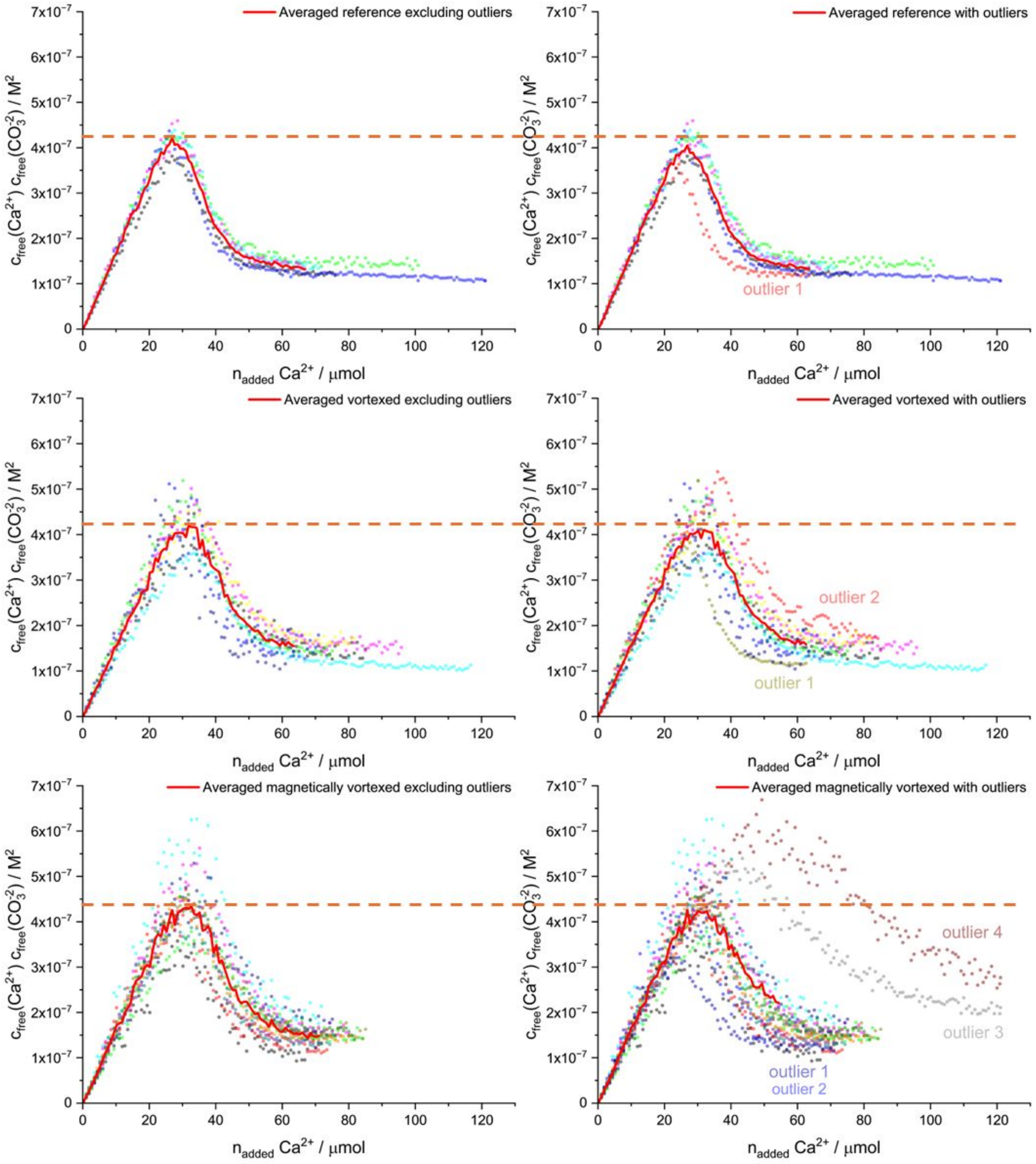

Figure SI 2. The ion products free calcium and carbonate ion concentrations against the amount of added $CaCl_2$ at pH 10 for the three conditions of reference, vortexed, and magnetically vortexed. The curves represented with dot symbols are the replicates, and the solid red line curve is averaged over the common X-axis for the three conditions, considering the outliers affecting them. The outliers on the right-handed graphs are labeled within each condition..

II) Scanning Electron Microscopy (SEM) and Attenuated Total Reflection Fourier-Transform Infrared (ATR-FTIR) sample preparation

After the nucleation curve reached the plateau leveled with solubility level at the crystallization stage, the nucleation process continued for 15 minutes. Then, the solution was centrifuged at 4750 rpm for 10 minutes to separate and decant the supernatant part- Subsequently, the solid phase was mixed with absolute ethanol, centrifuged at 4750 rpm for 10 minutes, and washed with acetone twice before being left air-dried under fume hood. Three samples were prepared for each condition of titrations

for SEM (JSM-6480, JEOL) and ATR-FTIR (ALPHA II Platinum ATR, Bruker) analysis. For SEM, the powder obtained from the procedure was evenly distributed as a thin layer on a carbon stick coated with a gold layer, and the analysis was performed at an accelerating voltage of 6 kV. An adequate quantity of samples was placed on the monolithic diamond crystal for ATR-FTIR, and absorbance was measured between 400 and 4000 cm^{-1}.

The FTIR spectra for each condition were averaged over the three repetitions, and the intensity of this average spectrum was normalized.

III) ATR-FTIR precipitation analyses

The FTIR spectra of precipitates from reference, vortexed, and magnetically vortexed conditions of potentiometric titrations of calcium carbonate are shown in the following. The bands ν_1, ν_2, ν_3, and ν_4 in the fingerprint zone below 2000 cm^{-1} correspond to the symmetric stretching, out-of-plane bending, asymmetric stretching, and in-plane bending of carbonate ion vibrational modes, respectively, primarily indicating a calcite crystalline structure. The shift at 744 cm^{-1} suggests the presence of vaterite. The $\nu_{O\text{-}H}$ band represents O-H stretching in water molecules, indicating water content. No changes in the spectrum were detected after pre-treatments, suggesting that the nanobubbles exist inherently and that treatments affecting their population do not alter the structure, unlike external forces.

The FTIR spectra gathered from the three examined groups are presented in Fig. SI3.

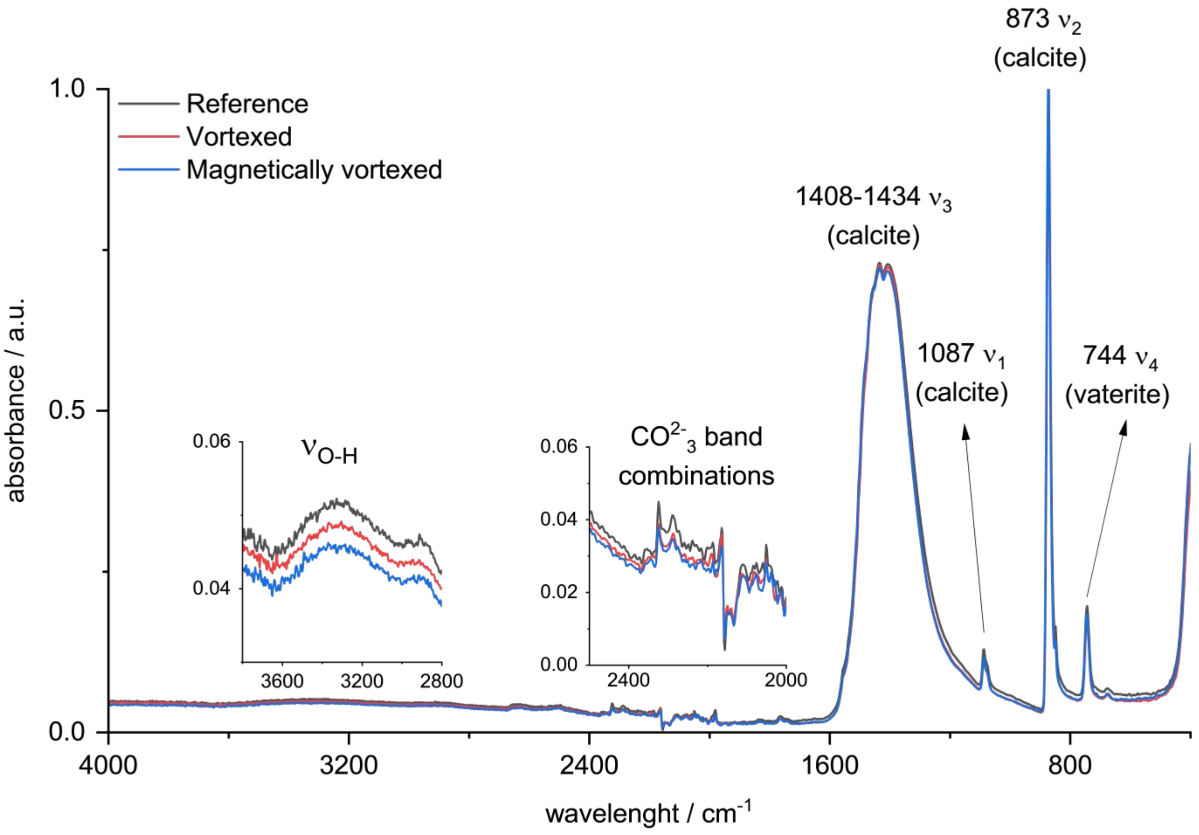

Figure SI 3. FTIR spectra of precipitations collected from three conditions of control, non-magnetic and magnetic impeller of potentiometric titrations of calcium carbonate.

IV) SEM precipitation analyses

SEM images at 5000x and 10000x magnifications of the three precipitation conditions (reference, vortexed, and magnetically vortexed) corroborate results obtained by ATR-FTIR analysis. As expected at pH 10, the dominant structure is calcite for all three experimental conditions. These results also align with the data on free Ca^{2+} concentration in the post-nucleation region, which showed no significant change, as shown in Figure 3 in the main manuscript.

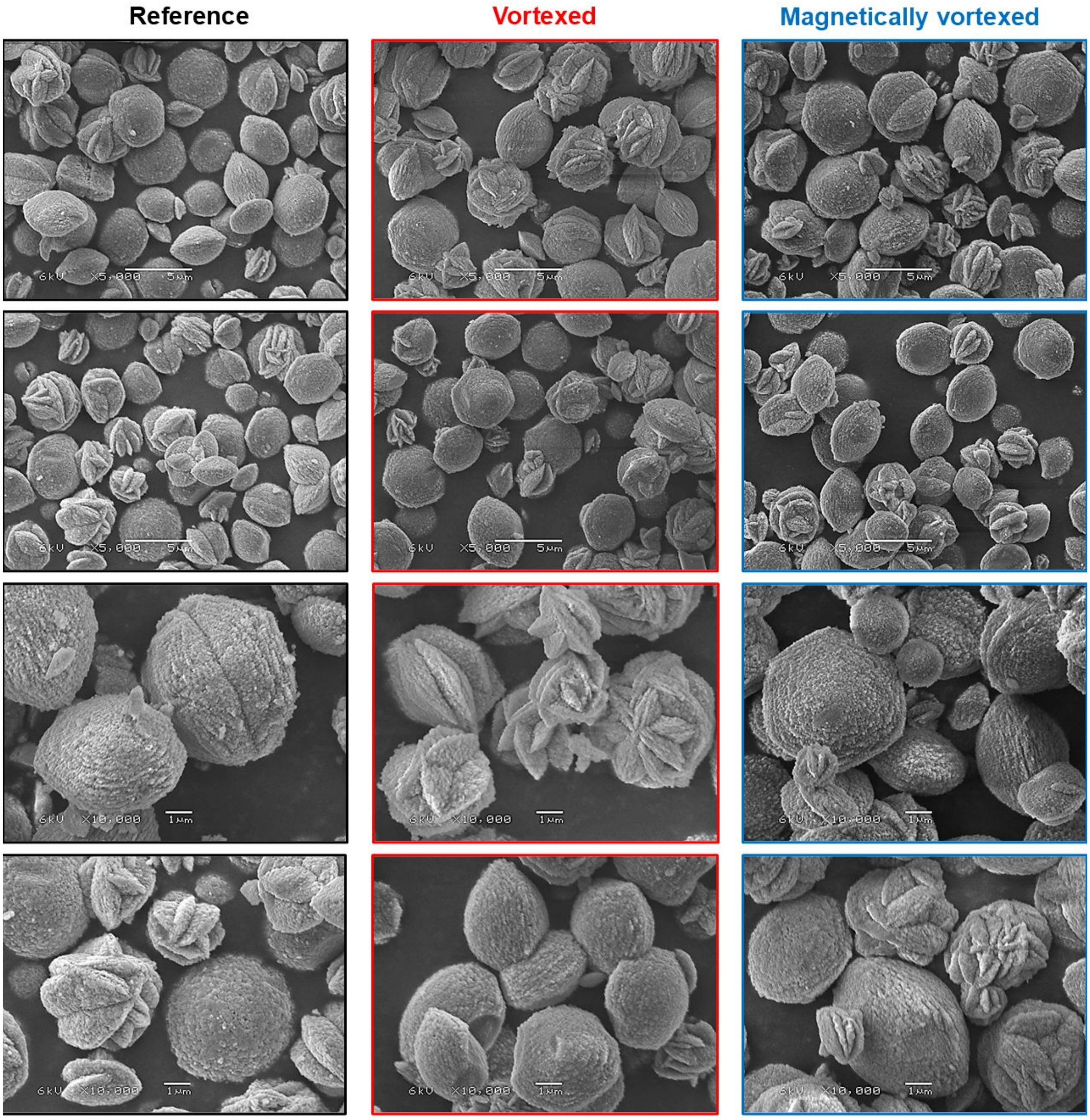

Figure SI 4. SEM images taken at two magnifications of 5000x and 10000x for the three conditions of precipitations

v) The hydrodynamic diameter extracted from Z-NTA measurements

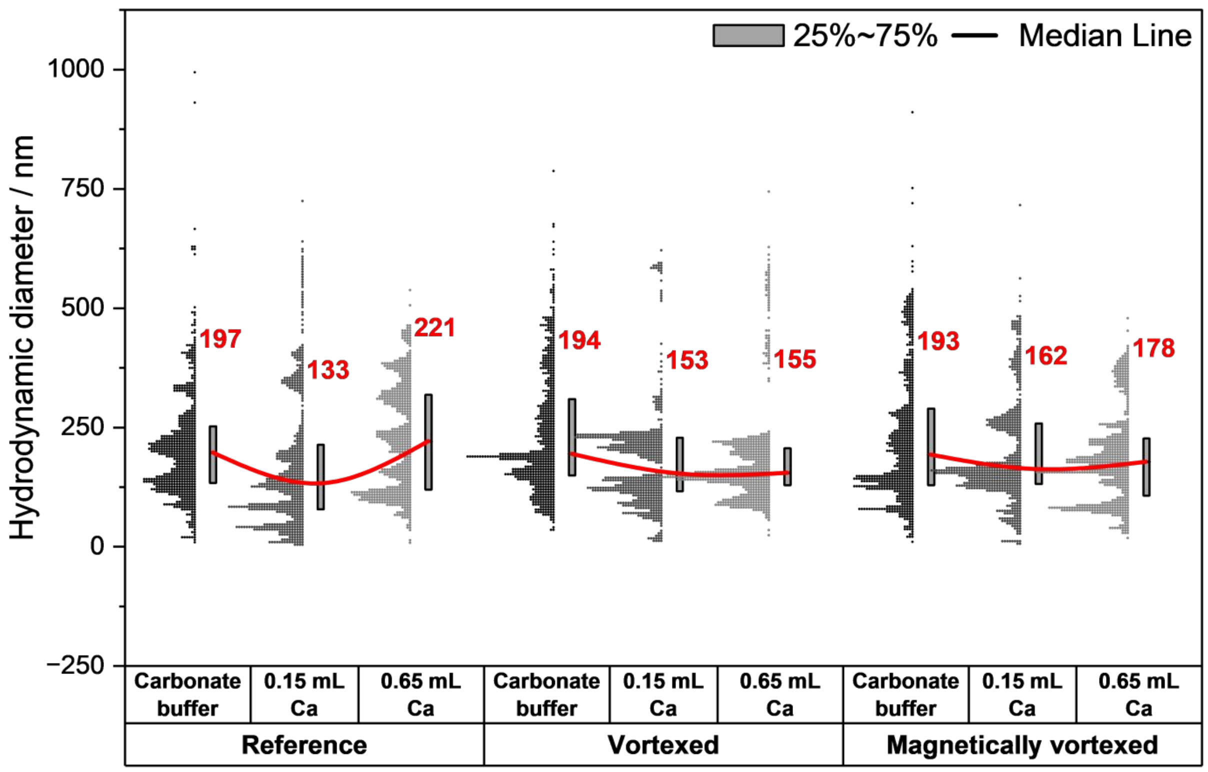

Figure SI 5. The hydrodynamic diameter of the three experimental groups of reference, vortexed and magnetically vortexed from the carbonate buffer, point 1 as the undersaturated sample after the addition of 0.15 mL of Ca, and point 3 as the supersaturated sample after the addition of 0.65 mL of Ca to the carbonate buffer. The solid red line connects the medians from each collection point of hydrodynamic diameter distributions to show the trend in the evolution of nanobubbles' zeta potential from carbonate buffer toward the nucleation, implicating almost no change detected.

The hydrodynamic diameter evolution shows a similar trend for all three experimental groups; however, the reference sample in the undersaturated stage exhibits slightly larger hydrodynamic diameters compared to the treated solutions. This difference may indicate that the nanobubbles in the reference have a slightly larger overall size or different surface characteristics. While the FTIR absorbance spectra show a subtle increase in the intensity of the ν_{O-H} peak (O–H stretching in water molecules) for the reference sample, suggesting higher water content, we cannot conclusively attribute the observed hydrodynamic diameter differences to variations in the hydration shell based solely on Z-NTA measurements. Instead, these observations may reflect changes in the nanobubble size or surface properties induced by the treatments. Further investigations using complementary techniques would be necessary to determine the role of the hydration shell in these systems.
